# *International Journal of Molecular Science* 2015’s Best Paper Award

**DOI:** 10.3390/ijms16023700

**Published:** 2015-02-06

**Authors:** Editorial Board of International Journal of Molecular Science

The* International Journal of Molecular Science* has previously granted [1,2,3], and is continuing with our practice of granting, annual awards to recognize outstanding papers in the area of chemistry, molecular physics and molecular biology published in its journal.

We are therefore pleased to announce the “*International Journal of Molecular Science* Best Paper Award” for 2015. Nominations were made by the Editorial Board Members chosen from all papers published in 2011. The awards are issued to reviews and research articles separately. We proudly reveal the following eight papers that have been chosen:

**Research Article Award:**

*First Prize*

**Toni Safner, Mark P. Miller, Brad H. McRae, Marie-Josée Fortin and Stéphanie Manel**

Comparison of Bayesian Clustering and Edge Detection Methods for Inferring Boundaries in Landscape Genetics

*Int. J. Mol. Sci.* **2011**, *12*(2), 865–889; doi:10.3390/ijms12020865

Available online: http://www.mdpi.com/1422-0067/12/2/865

“This study evaluates different techniques available for identifying clusters and boundary detection within populations. It nicely demonstrates the potential of Bayesian spatial clustering algorithms.”

*—Dr. Guido R. M. M. Haenen*

“While somewhat specialized, this study can have valuable practical utility in understanding where geographic edges lie between subspecies.”

*—Prof. Dr. Stephen C. Bondy*

*“The sheer breadth of the data, significance of conclusions and usefulness of results to other researchers.”*

*—Prof. Dr. Nicholas Delihas*

*Second Prize*

**Thomas Schlorf, Manuela Meincke, Elke Kossel, Claus-Christian Glüer, Olav Jansen and Rolf Mentlein**

Biological Properties of Iron Oxide Nanoparticles for Cellular and Molecular Magnetic Resonance Imaging

*Int. J. Mol. Sci.* **2011**, *12*(1), 12–23; doi:10.3390/ijms12010012

Available online: http://www.mdpi.com/1422-0067/12/1/12

*“The article investigated the uptake of different Iron oxide nanoparticles (wrt size, composition, coating), retention and location in the cell, and their ability to be detected by MRI. This paper was well written and will be of use to people working in the area of metal oxide nanoparticle research.”*

*—Dr. Terrence Piva*

*“Usefulness of results to MRI research and possibly clinical uses.”*

*—Prof. Dr. Nicholas Delihas*

**Juan C. Garro Martinez, Pablo R. Duchowicz, Mario R. Estrada, Graciela N. Zamarbide and Eduardo A. Castro**

QSAR Study and Molecular Design of Open-Chain Enaminones as Anticonvulsant Agents

*Int. J. Mol. Sci.* **2011**, *12*(12), 9354–9368; doi:10.3390/ijms12129354

Available online: http://www.mdpi.com/1422-0067/12/12/9354

*“This paper presents an interesting development of models for prediction of antiepileptic activity of some enaminones.”*

*—Prof. Dr. Fabio Marinelli*

*“The paper inscribes in the nowadays open issue of modeling ringed vs. open-chain molecular behavior, intuitively yet also computationally emphasizing on the last instance, in binding with receptor sites towards reliable mechanistic QSARs.”*

*—Dr. Mihai V. Putz*

*“Of use to researchers and good data, i.e., ED50 exp. vs. ED50 pred Figures. 3,4”*

*—Prof. Dr. Nicholas Delihas*

*Third Prize*

**Haider Raza, Subbuswamy K. Prabu, Annie John and Narayan G. Avadhani**

Impaired Mitochondrial Respiratory Functions and Oxidative Stress in Streptozotocin-Induced Diabetic Rats

*Int. J. Mol. Sci.* **2011**, *12*(5), 3133–3147; doi:10.3390/ijms12053133

Available online: http://www.mdpi.com/1422-0067/12/5/3133

*“May contribute to better understand the etiology of diabetes and may lead to development of new therapeutic methods.”*

*—Prof. Dr. Nicholas Delihas*

**Antonio Iannitelli, Rossella Grande, Antonio Di Stefano, Mara Di Giulio, Piera Sozio, Lucinda Janete Bessa, Sara Laserra, Cecilia Paolini, Feliciano Protasi and Luigina Cellini**

Potential Antibacterial Activity of Carvacrol-Loaded Poly(dl-lactide-*co*-glycolide) (PLGA) Nanoparticles against Microbial Biofilm

*Int. J. Mol. Sci.* **2011**, *12*(8), 5039–5051; doi:10.3390/ijms12085039

Available online: http://www.mdpi.com/1422-0067/12/8/5039

*“This paper presents an innovative nanoparticle-based approach to disrupting biofilms, a major challenge in biomedicine.”*

*—Prof. Dr.Vince Rotello*

**Review Award:**

**Alba Fernández-Sánchez, Eduardo Madrigal-Santillán, Mirandeli Bautista, Jaime Esquivel-Soto, Ángel Morales-González, Cesar Esquivel-Chirino, Irene Durante-Montiel, Graciela Sánchez-Rivera, Carmen Valadez-Vega and José A. Morales-González**

Inflammation, Oxidative Stress, and Obesity

*Int. J. Mol. Sci.* **2011**, *12*(5), 3117–3132; doi:10.3390/ijms12053117

Available online: http://www.mdpi.com/1422-0067/12/5/3117

*“A very comprehensive review on obesity, an inflammatory condition which represents a major risk factor for a number of chronic diseases, including diabetes, cardiovascular diseases and cancer.”*

*—Prof. Dr. Marcello Iriti*

*“This is a very important topic, the authors did a nice job in reviewing it in an interesting way”*

*—Dr. William Chi-shing Cho*

*“This is a comprehensive review of the literature on obesity especially in regards the effects adipokines play in regards modulating oxidative stress and immunity in the host. This review article is one that will be used as a reference source for those working in obesity research.”*

*—Dr. Terrence Piva*

*“This Review presents a very well-considered discussion of the complex interrelationship of obesity and inflammation. An excellent starting point for anyone interested in the underlying causes of obesity-related inflammation.”*

*—Prof. Dr. Vince Rotello*

*“This Review provides a very clear and thorough overview of an area of research that is increasingly important. The authors convey the multifactorial nature of the factors that influence obesity and the review helps explain how our lifestyle is intimately linked with the obesity problem in industrialised countries and why weight loss is so difficult.”*

*—Prof. Dr. Stephen A. Bustin*

*“Good descriptions of factors related to obesity in Abstract, Fig. 1 and Conclusions and weight loss with nutritional and drug treatments.”*

*—Prof. Dr. Nicholas Delihas*

**Maryam Moravej and Diego Mantovani**

Biodegradable Metals for Cardiovascular Stent Application: Interests and New Opportunities

*Int. J. Mol. Sci.* **2011**, *12*(7), 4250–4270; doi:10.3390/ijms12074250

Available online: http://www.mdpi.com/1422-0067/12/7/4250

*“This review that describes the recent advances in biodegradable metallic stents, is of high quality and high impact.”*

*—Prof. Dr. Fabio Marinelli*

“This review gives a relatively complete overview of the recent developments in metallic materials for biodegradable stents.”

*—Dr. Guido R. M. M. Haenen*

*“This study on biodegradable stents is well-written annd has the potential for wide applicability. It is of broad relevance and may lead to very useful advances in medical science.”*

*—Prof. Dr. Stephen C. Bondy*

*“Fascinating discussion of biodegradable cardiovascular stents. But I am surprised at the lack of toxicity (in animals) and the degradation rate needs to be worked out.”*

*—Prof. Dr. Nicholas Delihas*

**Giuseppe Vasapollo, Roberta Del Sole, Lucia Mergola, Maria Rosaria Lazzoi, Anna Scardino, Sonia Scorrano and Giuseppe Mele**

Molecularly Imprinted Polymers: Present and Future Prospective

*Int. J. Mol. Sci.* **2011**, *12*(9), 5908–5945; doi:10.3390/ijms12095908

Available online: http://www.mdpi.com/1422-0067/12/9/5908

*“This review is very interesting, because the topic covered is of great importance. It is written in a clear manner, and the citation of references is exhaustive.”*

*—Prof. Dr. Fabio Marinelli*

*“This is a forward-looking review in an area likely to develop a lot in the next few years.”*

*—Prof. Dr. Stephen C. Bondy*

*“Molecular imprinting technology seems throughly described and presented.”*

*—Prof. Dr. Nicholas Delihas*

We believe that these eight exceptional papers are valuable contributions to the *International Journal of Molecular Science* and the scientific research field. On behalf of the Prize Awarding Committee and the Editorial Board of *International Journal of Molecular Science*, we would like to congratulate these teams for their excellent work. In recognition of their accomplishment, Dr. Stéphanie Manel, Dr. Rolf Mentlein, Dr. Juan C. Garro Martinez, Dr. Haider Raza and Dr. Antonio Di Stefano will receive a prize of 1000 CHF, 600 CHF, 600 CHF, 400 CHF and 400 CHF, respectively, and the privilege of publishing an additional paper free of charge in open access format in the *International Journal of Molecular Science*, after the usual peer-review procedure. Dr. José A. Morales-González, Dr. Diego Mantovani and Dr. Giuseppe Vasapollo will receive the privilege of publishing an additional research article free of charge in open access format in the *International Journal of Molecular Science*, after the usual peer-review procedure.

**Prize Awarding Committee**

Prof. Dr. Anthony LemariéProf. Dr. Nicholas DelihasProf. Dr. Fabio MarinelliProf. Dr. Pamela LeinDr. Guido R. M. M. HaenenProf. Dr. Paul EvansProf. Dr. Helmut SegnerProf. Dr. Stephen C. BondyProf. Dr. Ian A. NichollsDr. Terrence PivaProf. Dr. Kaixun HuangProf. Dr. Vince RotelloProf. Dr. Kurt A. JellingerProf. Dr. Vladimír KřenDr. Lam-Son Phan TranDr. Walter HerzogProf. Dr. Marcello IritiDr. William Chi-shing ChoDr. Marie-Christine BacchusDr. Xiaofeng JiaDr. Mateus Webba da SilvaProf. Dr. Zdenek WimmerDr. Mihai V. Putz
